# Meteorological Observations for April, 1857, at Atlanta, Ga.

**Published:** 1857-06

**Authors:** J. G. Westmoreland


### METEOROLOGICAL OBSERVATIONS FOR APRIL, 1857, AT ATLANTA, GA.

| APRIL. | THERMOMETER. | | | BAROMETER. | | | WIND. | REMARKS. |
|---|---|---|---|---|---|---|---|---|
| | 7 A. M. | 2 P. M. | 7 P. M. | 7 A. M. | 2 P. M. | 7 P. M. | | |
| 1 | 50 | 64 | 52 | 29.77 | 29.75 | 29.70 | S. | Cloudy—Rain ½ in. |
| 2 | 40 | 56 | 46 | 29.72 | 29.90 | 29.94 | N. W. | Fair. |
| 3 | 36 | 54 | 44 | 30.07 | 30.20 | 30.10 | N. W. | Fair. |
| 4 | 44 | 64 | 58 | 30.10 | 30.10 | 30.00 | S. | Cloudy—Rain 9-16 in. |
| 5 | 54 | 60 | 58 | 29.90 | 29 85 | 29.70 | W. | Drizzly. |
| 6 | 28 | 34 | 28 | 29.62 | 29.75 | 29.77 | N. W. | Fair. |
| 7 | 28 | 48 | 42 | 29.90 | 30.06 | 30.00 | W. | Fair. |
| 8 | 38 | 62 | 52 | 30.02 | 30.05 | 30.00 | S. W. | Fair. |
| 9 | 44 | 44 | 37 | 29.85 | 29.85 | 29.80 | W. | Cloudy—Rain ¼. |
| 10 | 34 | 56 | 48 | 29.72 | 29.85 | 29.75 | N. W. | Fair. |
| 11 | 42 | 60 | 46 | 29.72 | 29.85 | 29.75 | W. | Fair. |
| 12 | 34 | 38 | 38 | 29.70 | 29.70 | 29.70 | E. | Cloudy—Rain ⅛. |
| 13 | 34 | 58 | 50 | 29.70 | 29.75 | 29.80 | W. | Fair. |
| 14 | 38 | 48 | 40 | 29.75 | 29.90 | 29 87 | W. | Cloudy—Drizzly. |
| 15 | 42 | 64 | 44 | 29.80 | 29.80 | 29.75 | W. | Hazy. |
| 16 | 38 | 45 | 42 | 29.70 | 29.80 | 29.75 | W. | Hazy. |
| 17 | 48 | 50 | 55 | 29.90 | 29.85 | 29.75 | W. | Fair. |
| 18 | 60 | 70 | 58 | 29.73 | 29.73 | 29.65 | S. W. | Cloudy, Storm, R'n ¾ in. |
| 19 | 58 | 44 | 44 | 29.70 | 29.75 | 29.80 | W. | Cloudy—Rain 3-16. |
| 20 | 32 | 60 | 48 | 29.90 | 29.85 | 29.85 | N W. | Fair. |
| 21 | 44 | 64 | 42 | 29.70 | 29.62 | 29.68 | W. | Hazy—Windy. |
| 22 | 38 | 44 | 40 | 29.65 | 29 90 | 29.80 | W. | Hazy. |
| 23 | 32 | 52 | 52 | 29.80 | 29 90 | 29.90 | W. | Fair. |
| 24 | 42 | 48 | 42 | 29.90 | 29.92 | 29.95 | W. | Fair. |
| 25 | 42 | 64 | 58 | 29.92 | 30.05 | 29.91 | W. | Fair. |
| 26 | 50 | 70 | 64 | 29 90 | 29.90 | 29.80 | S. | Cloudy—Rain ¼. |
| 27 | 52 | 62 | 54 | 29.80 | 29.90 | 29.90 | N. W. | Flying Clouds. |
| 28 | 52 | 70 | 58 | 29.92 | 29.95 | 29.95 | W. | Hazy. |
| 29 | 50 | 68 | 66 | 29.95 | 30.05 | 30.00 | W. | Fair. |
| 30 | 60 | 72 | 66 | 30.08 | 30.08 | 29.95 | N. W | Fair. |

Furnished by

J. G. WESTMORELAND, M. D.

